# Magnetic resonance imaging negative myelopathy in Leber’s hereditary optic neuropathy: a case report

**DOI:** 10.1186/s12883-022-03007-3

**Published:** 2022-12-15

**Authors:** Mika H. Martikainen, Miika Suomela, Kari Majamaa

**Affiliations:** 1 Clinical Neurosciences, Department of Clinical Medicine, University of Turku, Turku, Finland; 2 Neurocenter, Turku University Hospital, Turku, Finland; 3 Department of Neurology, Satasairaala Hospital, Pori, Finland; 4 Department of Clinical Neurophysiology, Turku University Hospital, Turku, Finland; 5 Research Unit of Clinical Neuroscience, Neurology, University of Oulu, Oulu, Finland; 6 Neurocenter and Medical Research Center, Oulu University Hospital, Oulu, Finland

**Keywords:** Case report, Leber hereditary optic neuropathy (LHON), Myelopathy, Mitochondrial disease, Mitochondrial DNA (mtDNA), Somatosensory evoked potential (SEP)

## Abstract

**Background:**

Leber’s hereditary optic neuropathy (LHON) is a common form of mitochondrial disease. The typical clinical presentation of LHON is subacute, painless loss of vision resulting from bilateral optic nerve atrophy. Moreover, extra-ocular manifestations such as cardiac conduction abnormalities and neurological manifestations such as multiple sclerosis (MS) like disease or parkinsonism are encountered in some patients. Abnormal findings in spinal cord MR imaging or in the cerebrospinal fluid (CSF) have been observed in previous cases of LHON-associated myelopathy.

**Case presentation:**

We report a male patient with LHON who developed symptoms of myelopathy including gait unsteadiness, enhanced deep tendon reflexes and sensory loss of the lower extremities. Imaging of the brain and spinal cord, CSF analysis, as well as neurography and electromyography did not disclose any abnormalities. The somatosensory evoked potential (SEP) findings were suggestive of dorsal column dysfunction.

**Conclusions:**

The patient case demonstrates that myelopathy associated with LHON can present without abnormal findings in central nervous system MR imaging or in the CSF, and without evidence suggestive of multiple sclerosis or MS-like disease. The dorsal column seems to be particularly vulnerable to myelopathy changes in LHON. Evoked potential investigations may assist in confirming the diagnosis, when clinical features are in line with myelopathy but findings in CSF analysis and central nervous system imaging are normal.

## Background

Leber’s hereditary optic neuropathy (LHON, OMIM 535000) is a common mitochondrial disease, affecting about 1:31,000 to 1:54,000 individuals in European populations [1]. The typical clinical presentation of LHON is subacute, painless loss of vision resulting from bilateral optic nerve atrophy. The disease onset is typically in adult age, with both eyes affected simultaneously or in few months’ succession [2]. Moreover, extra-ocular manifestations such as cardiac conduction abnormalities [3] and neurological manifestations such as dystonia, tremor, and parkinsonism [4] are encountered in some patients. Association of LHON with multiple sclerosis (MS) has also been reported and multiple sclerosis –like disease has been described with multiple episodes of visual impairment, predominance for women, and an average interval of 1.66 years in the impairment of the eyes [5, 6].

Here we report a male LHON patient of Finnish descent. He developed symptoms of myelopathy including gait unsteadiness, enhanced deep tendon reflexes as well as pain and sensory loss of the lower extremities during the disease course, but findings in MR imaging of the brain and spinal cord and in CSF analysis were normal. Evoked potential investigations provided evidence for myelopathy.

## Case presentation

The patient is a 51-year-old man who presented to ophthalmologist at the age of 46 years because of progressive bilateral visual loss during the previous 3 months. Clinical findings and history suggested LHON that was confirmed by genetic testing. He was a smoker. At age 47, he had visual acuity below 0.1 bilaterally, could count fingers and was able to move about independently. He started taking idebenone 300 mg tds at age 47, but this medication was discontinued after 1 year because of lack of efficacy.

He was referred to neurologist at age 47 because of unsteady and broad-based gait, inability to walk a straight line, lower limb pain and sensory loss. Neurological examination disclosed that both patellar and ankle jerk reflexes were bilaterally enhanced but without clonus and plantar responses were in flexion. The muscle tone was normal. Gait was broad-based, and there was difficulty in tandem gait. Light touch and pinprick sensation were diminished, vibration sensation was absent, and he reported tingling and numbness below the knee level (L5). At follow-up visits at ages 48 to 51, the patient was still independently ambulatory but used a white cane. Unassisted gait was unsteady and broad-based, and he could not perform tandem gait. He now reported sensory abnormalities up to the groin level (L1). Subtle action tremor of both hands was observed, but the examination did not suggest an extrapyramidal or cerebellar disorder. Eye movements, speech, and cognition were normal. Bladder function was normal.

### Investigations

**Fig. 1:**
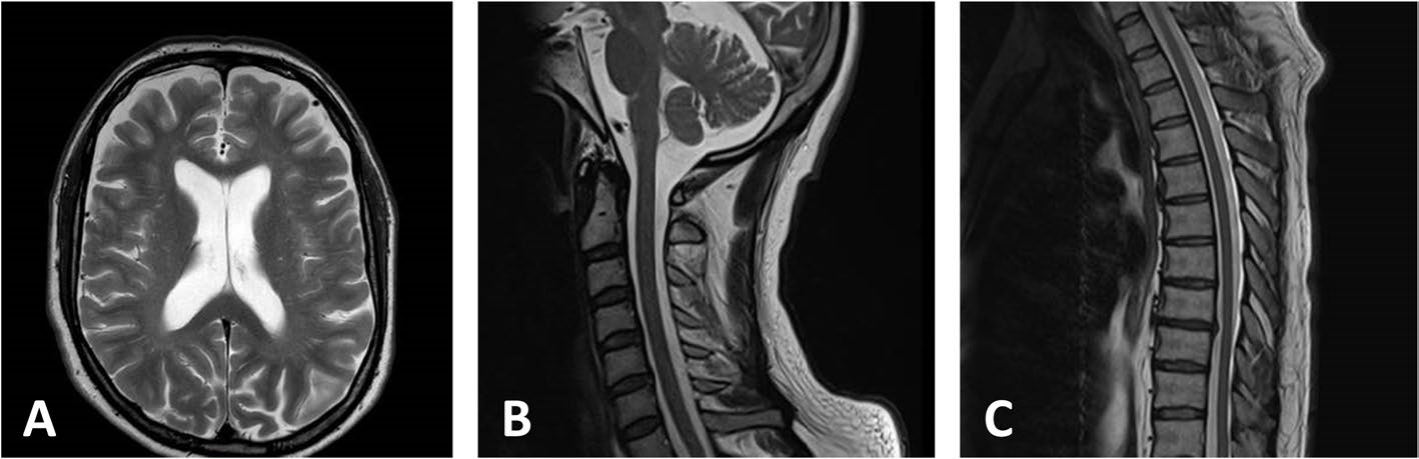
MR imaging findings. MR imaging of the brain and spinal cord did not reveal findings suggestive of myelopathy or multiple sclerosis. **A**. brain, **B**. cervical spinal cord, **C**. thoracic spinal cord. All shown MR images are T2 weighted

Molecular genetic testing revealed homoplasmic m.11778G > A variant in mtDNA, confirming the diagnosis of LHON. His mother harboured homoplasmic m.11778G > A, whereas the two siblings of his mother and his maternal grandmother were not examined. These maternal relatives were reported to be unaffected. At age 47, brain MR imaging revealed a non-expansive arachnoid cyst in the posterior fossa but was otherwise normal and MR imaging of the spinal cord was normal (Fig. 1). Laboratory work-up did not reveal any abnormalities that the symptoms could be attributed to. Fasting blood glucose (5.6 mmol/l) and HbA1c (37 mmol/mol; reference 20–42 mmol/mol) were normal. Serum vitamins B_1_ (188 nmol/l; reference 60–230 nmol/l), B_12_ (105 pmol/l; reference > 35 pmol/l) as well as B_9_ (folate) (648 nmol/l; reference 285-1475 nmol/l) were normal. In CSF, protein was mildly elevated (440 mg/l; reference range 105–290 mg/l), but there was no pleocytosis, and there were no oligoclonal immunoglobulin G bands. There were no antibodies against *Borrelia burgdorferi* in either serum or CSF.

**Fig. 2:**
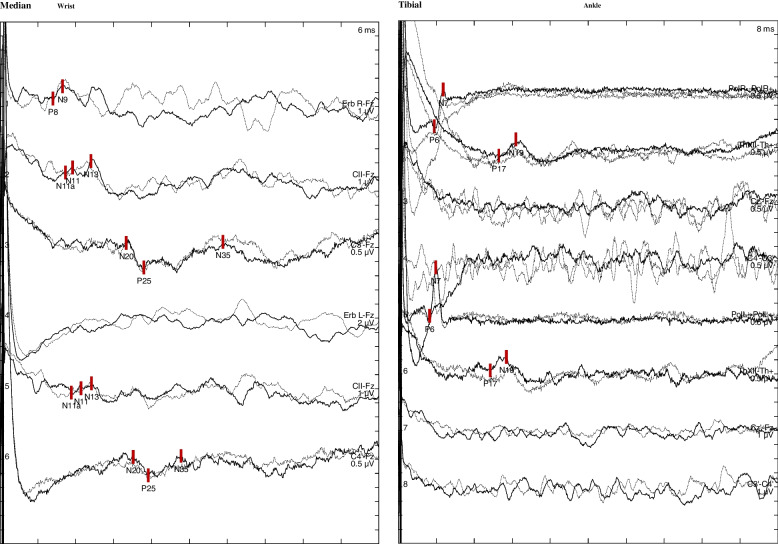
Findings in the SEP investigation. Left panel: Median nerve SEP. N13 latencies 14.5 ms on both sides (upper bound of 97.5% confidence interval (CI); 13.1 ms (right), 12.8 ms (left). N20-latencies 20.0 ms (right) and 21.1 ms (left) (upper bound of CI; 19.2 ms (right) and 18.8 ms (left)). Right panel: Tibial nerve SEP. Normal peripheral and lumbar potentials but absent cortical potentials. SEP = sensory evoked potential

Neurography and electromyography at age 47 were normal. Investigation of somatosensory evoked potentials (SEP) at age 48 showed that cortical responses to tibial nerve stimulation were absent, whereas the lumbar responses were normal. Cortical N20 response to median nerve stimulation was bilaterally abnormal with increased latency and distorted wave morphology. The latency of N13 response was prolonged too (Fig. 2). Motor evoked potentials using transcranial magnetic stimulation were normal.

## Discussion and conclusions

Our patient harboured m.11778G > A, the most common mtDNA variant associated with LHON [7]. He experienced the loss of vision typical for the disease at age 46 and developed myelopathy symptoms within 2 years thereafter. MR imaging of the central nervous system and CSF investigation showed normal results, but the findings in evoked potentials investigation were suggestive of posterior column dysfunction, either at cervical level or reflecting a more diffuse posterior column dysfunction.

There are previous reports of myelitis or myelopathy in patients with LHON [8–12]. In these cases, however, MR imaging of the spinal cord has been abnormal or signs of inflammation or autoimmune activation have been observed in the CSF. In most cases, the tone of the lower limb muscles has been reported to be increased. Autopsy studies of LHON patients have revealed spinal cord degeneration particularly in the posterior column and posterior spinal roots, but Leigh-like subacute infarction of the spinal cord has also been reported [9]. Serum copper levels were not measured. Copper deficiency myelopathy would be an unlikely diagnosis as the patient was male, there was no anaemia or other cytopenia, and spinal cord MR imaging was normal [13]. The patient was not tested for syphilis, which is today rare in Finland. There was no medical history of syphilis, and there was no pleocytosis in the CSF. Moreover, several features suggestive of tabes dorsalis, such as Argyll Robertson pupils, lancinating limb pain, and Charcot joints, were not present [14].

Despite clinical examination and MR imaging, the conclusive diagnosis or exclusion of myelopathy is sometimes difficult. Evoked potential studies are sensitive tools to detect even subtle central nervous system lesions. Standard SEP technique assesses mainly the function of the posterior column–lemniscal system [15]. Abnormalities of evoked responses reflect the global damage of the evoked nervous pathway and are more sensitive than MR imaging to reveal spinal cord lesions in MS [16]; SEP investigation is also more sensitive than spinal cord MR imaging in detecting subacute combined degeneration of the spinal cord caused by vitamin B_12_ deficiency [17]. In a previous study, abnormal findings in both MEP and SEP studies were common in patients with various types of mitochondrial disease, but no data on LHON patients were included [18].

LHON epidemiology has been previously studied in several countries, including Finland. The prevalence of LHON has been reported to be 2.0/100,000 in Finland [19] and 3.7/100,000 in the North East of England [20]. In the Finnish study, the penetrance was 31% among men and 8% among women in families with the homoplasmic m.11778G > A mutation, but it was highly variable between families [19].

Even though myelopathy is uncommon in LHON and the reported cases remain sparse, the link between LHON and the development of myelopathy is probable. The development of myelopathy in LHON is plausibly related to the respiratory chain dysfunction in mitochondrial disease, and the dorsal column seems to be particularly vulnerable [9, 12]. Our findings suggest that evoked potential investigations may assist in confirming the diagnosis, when clinical features are in line with possible myelopathy but findings in CSF analysis and central nervous system imaging are normal.

## Data Availability

Not applicable.

## Abbreviations

CSF: Cerebrospinal fluid
LHON: Leber hereditary optic neuropathy
MEP: Motor evoked potential
MR: Magnetic resonance
MS: Multiple sclerosis
mtDNA: Mitochondrial deoxyribonucleic acid
SEP: Sensory evoked potential
